# DNA methylation in the *OPG*/*RANK*/*RANKL* pathway is associated with steroid-induced osteonecrosis of the femoral head

**DOI:** 10.1186/s12891-021-04472-6

**Published:** 2021-06-29

**Authors:** Menghu Sun, Yuju Cao, Xiaolong Yang, Feimeng An, Huiqiang Wu, Jianzhong Wang

**Affiliations:** 1 Department of Orthopedics and Traumatology, The Second Affiliated Hospital of Inner Mongolia Medical University, Hohhot, 010030 Inner Mongolia China; 2 Inner Mongolia Medical University, Hohhot, 010050 Inner Mongolia China; 3 Zhengzhou Traditional Chinese Medicine (TCM) Traumatology Hospital, Zhengzhou, 450016 Henan Province China; 4 Inner Mongolia Autonomous Region Hospital of Traditional Chinese Medicine, Hohhot, 010110 Inner Mongolia China

**Keywords:** *OPG*, *RANK*, *RANKL*, Methylation, Steroid-induced ONFH

## Abstract

**Background:**

Dysregulation of the *OPG*/*RANK*/*RANKL* signalling pathway is a key step in the occurrence of steroid-induced osteonecrosis of the femoral head (ONFH). This study aims to understand the degree of methylation of the *OPG*, *RANK*, and *RANKL* genes in steroid-related ONFH.

**Methods:**

A case-control study was designed, including 50 patients (25 males and 25 females) and 50 matched controls. The European Molecular Biology Open Software Suite (EMBOSS) was used to predict the existence and location of CpG islands in the *OPG*, *RANK*, and *RANKL* genes. The Agena MassARRAY platform was used to detect the methylation status of the above genes in the blood of subjects. The relationship between the methylation level of CpG sites in each gene and steroid-related ONFH was analysed by the chi-square test, logistic regression analysis, and other statistical methods.

**Results:**

In the CpG islands of the *OPG*, *RANK*, and *RANKL* genes in patients with steroid-related ONFH, several CpG sites with high methylation rates and high methylation levels were found. Some hypermethylated CpG sites increase the risk of steroid-related ONFH. In addition, a few hypermethylated CpG sites have predictive value for the early diagnosis of steroid-related ONFH.

**Conclusion:**

Methylation of certain sites in the *OPG*/*RANK*/*RANKL* signalling pathway increases the risk of steroid-related ONFH. Some hypermethylated CpG sites may be used as early prediction and diagnostic targets for steroid-related ONFH.

## Background

Steroid-induced osteonecrosis of the femoral head (steroid-induced ONFH) is a disease primarily due to the extensive use of hormones. It can lead to blood circulation disorders of the femoral head, bone cell ischaemia, degeneration, necrosis, irreversible collapse of the femoral head, and ultimately cause hip joint dysfunction [1]. According to reports, the incidence of steroid-induced ONFH is increasing every year, and it is one of the most common non-traumatic ONFHs, accounting for approximately 24.1% of the total number of patients with ONFH [2]. However, the early diagnosis of this disease is difficult, and it often progresses to the late stage, requiring hip replacement surgery, which seriously affects the quality of life of patients [3]. Therefore, the early diagnosis and treatment of the disease is the focus of current research.

The pathological process of steroid-induced ONFH is complicated. In the past, numerous scholars have proposed various theories for this, including lipid abnormalities, endothelial cell damage, coagulation defects, genetic polymorphism, apoptosis, autophagy, and so on [4–6]. In recent years, with the development of molecular biology and genetics, epigenetics, which studies the interaction between environmental signals and genomes, has received increasing attention. Among epigenetics, DNA methylation is undoubtedly one of the most important fields. Studies have shown that DNA methylation plays a role in the occurrence and development of ONFH [7–9]. However, none of these alone can fully explain its underlying mechanism.

Although the cause is not clear, abnormal bone metabolism is a key step in the occurrence of steroid-induced ONFH, and the *OPG*/*RANK*/*RANKL* system is involved in this process [10]. Receptor activator of nuclear factor-κ B (*RANK*) is a member of the tumour necrosis factor receptor (TNFR) family, located on the surface of osteoclasts and osteoclast precursor cells. Its transduction receptor *RANKL* is a cytokine necessary for osteoclast differentiation and is synthesized by osteoblasts and bone marrow stromal cells. *RANK* can stimulate the differentiation and maturation of osteoclasts by binding to *RANKL* receptors. Osteoprotegerin (*OPG*) belongs to the TNFR superfamily and mainly secreted by osteoblasts in bone tissues. In addition, it is a natural inhibitor of *RANKL*. *OPG* can block the binding of *RANKL* and *RANK* by competitively binding to *RANKL*, thereby inhibiting the activity and maturation of bone cells. *OPG*, *RANK*, and *RANKL* together constitute the regulatory axis for osteoclasts [11]. It is currently believed that steroids mainly cause an imbalance in the bone reconstruction process of the femoral head through the *OPG*/*RANK*/*RANKL* signalling pathway, inhibit bone formation, and cause bone loss and osteonecrosis [12].

In this study, we used the Agena MassARRAY system to analyse the methylation status of the CpG islands of the *OPG*, *RANK*, and *RANKL* genes in the blood of patients with steroid-induced ONFH and normal control subjects. The purpose of this study was to determine whether methylation in the *OPG*/*RANK*/*RANKL* signalling pathway and the possible relationship between methylation levels and steroid-induced ONFH.

## Methods

### Subjects

A total of 100 subjects were recruited for this study, including 50 patients with steroid-induced ONFH and 50 healthy controls. All participants were from Zhengzhou Traditional Chinese Medicine (TCM) Traumatology Hospital in Henan Province. The case group was composed of patients who were diagnosed with steroid-induced ONFH during hospitalization and were classified using the latest Association Research Osteocirculatory System (ARCO) [13]. The control group consisted of healthy people who were examined at the same hospital’s physical examination centre during the same period and matched the age and sex of the case group. All participants were Han people from northern China who lived in or around Zhengzhou Province without any kinship.

To only consider the effect of methylation of the *OPG*, *RANK*, and *RANKL* genes on steroid-induced ONFH as much as possible, we set stricter inclusion and exclusion criteria based on some reports related to the risk factors for the disease. It is impossible to exclude all risk factors, which is why the EWAS and differential methylation methods such as Bumphunter were developed later. However, other unconsidered risk factors are random between the case and the control group.

The criteria for the cases included in this study were as follows: (1) ONFH patients with typical clinical manifestations confirmed by magnetic resonance imaging (MRI), computer tomography (CT) or X-ray [14]; (2) before the appearance of clinical manifestations of ONFH, patients had taken a dose equivalent to prednisolone with an average dose of 16.6 mg/day for one year. Or a maximum dose of 80 mg/day for more than a week [15]; (3) the patient should not have a history of trauma or other hip joint diseases; and (4) some other risk factors that may affect the results of the test were excluded (history of alcoholism [16]; family history of genetic diseases; and severe diseases or severe chronic diseases, such as cardiovascular and cerebrovascular diseases, diabetes, and chronic renal insufficiency).

The selection criteria for the controls were as follows: (1) not suffering from ONFH; (2) the age and sex of the control group matched the case group; and (3) certain other risk factors were excluded (such as congenital diseases, diabetes, cardiovascular disease, liver and kidney dysfunction, cancer, and family history of diseases).

### DNA extraction and methylation analysis

According to the manufacturer’s instructions, a whole blood genomic DNA extraction kit (GoldMag Co., Ltd., Xi’an, China) was used to extract total DNA. DNA quality was evaluated using a Nanodrop 2000 (Thermo Scientific, Waltham, Massachusetts, USA).

**Fig. 1:**
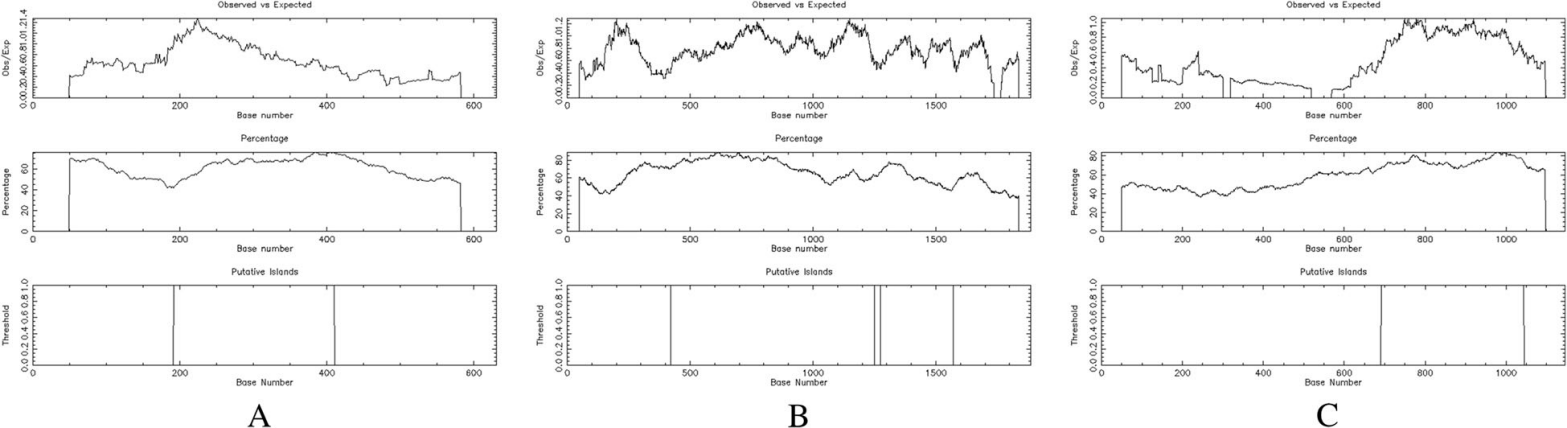
EMBOSS software predicted the CpG islands in the *OPG*, *RANK* and *RANKL* genes. A CpG island in the *OPG* gene sequence, with a length of 219 bp (**A**). Two CpG islands were found in the *RANK* gene sequence, one CpG island was 829 bp in length, and the other was 296 bp (**B**). A CpG island was found in the *RANKL* gene sequence, with a length of 354 bp (**C**)

The European Molecular Biology Open Software Suite (EMBOSS,

https://www.ebi.ac.uk/Tools/seqstats/emboss_cpgplot/) was used to analyze the existence and location of CpG islands in the *OPG*, *RANK*, and *RANKL* genes. We found a CpG island (chr8:118,952,332 ~ 118,952,550) in the *OPG* gene sequence, with a length of 219 bp (Fig. 1A). Two CpG islands (chr18:62,325,388 ~ 62,326,216 and 62,326,241 ~ 62,326,536) were found in the *RANK* gene sequence, one CpG island was 829 bp in length, and the other was 296 bp (Fig. 1B). A CpG island (chr13:42,574,105 ~ 42,574,458) was found in the *RANKL* gene sequence, with a length of 354 bp (Fig. 1C).

Based on base-specific cleavage and MALDI-TOF mass spectrometry, EpiTyper MassARRAY (Agena, San Diego, California, USA) was used for quantitative methylation analysis of the *OPG*, *RANK*, and *RANKL* genes. An EZ Bisulfite Conversion Kit (Zymo Research, Orange, California, USA) was used for bisulfite transformation of the *OPG*, *RANK*, and *RANKL* genes. Then, polymerase chain reaction (PCR) amplification was performed. The PCR primers used in this study were designed according to the results of EMBOSS and Epidesigner (http://www.epidesigner.com/) and were synthesized by Beijing Augct Biotechnology Co., Ltd. The primer sequences are shown in Table 1.

**Table 1:** Primers used for this study

Gene_ID	Direction	Primer (5′ → 3′)	Product	Number of
			Length (bp)	CG
*OPG*1	F	aggaagagagTTTTTGTTGTTTTTTATAAAGTTAGTAGGA	185	6
	R	cagtaatacgactcactatagggagaaggctACTACTACCACCTAATCTCCCAACC		
*OPG*2	F	aggaagagagGAAAGGTGTAAAGTTTGGTTTAGGA	265	21
	R	cagtaatacgactcactatagggagaaggctAAAAAACCAAATAACAACAACCTCC		
*RANK*1	F	aggaagagagAAGAAAAAGAGATAGTGGTTGTTGGT	464	33
	R	cagtaatacgactcactatagggagaaggctCAAATAATACCCAAACTCCCCTAAT		
*RANK*2	F	aggaagagagGGTTTTGATGTTGTTATTTTTTTTAAATGT	491	38
	R	cagtaatacgactcactatagggagaaggctCCTTCCCTATAAAAACTTTCAAATTC		
*RANK*3	F	aggaagagagATTTGAAAGTTTTTATAGGGAAGGG	349	12
	R	cagtaatacgactcactatagggagaaggctAAACACTTAATTAAACAACACCTAAAA		
*RANKL*1	F	aggaagagagTAGAGGTGGGAGTGGAAGAGGTAGTT	497	46
	R	cagtaatacgactcactatagggagaaggctATCCCCTAAAAAAATAACCACTCAC		
*RANKL*2	F	aggaagagagGATTTTTTGGGAAGGTGGTTATTTA	235	19
	R	cagtaatacgactcactatagggagaaggctCCAACAAAAACTACACCAAATACCT		

*F* Forward primer, *R* Reverse primer

### Statistical analyses

All statistical analyses in this study were performed using SPSS 24.0 software. The data are expressed as the mean ± standard deviation. The Shapiro-Wilk test was used to detect the normality of samples. We also used the non-parametric test and obtained the same conclusion. For samples that met the criteria, the two-sided t-test was selected when analysing the methylation level of genes between groups. Otherwise, the Mann-Whitney test was chosen. Pearson’s chi-square test was used to analyse the methylation rate of genes between groups. Logistic regression analysis was selected to analyse the relationship between the methylation level of genes and the risk of steroid-induced ONFH. ROC curves were used to determine the predictive diagnostic value of methylated genes for the disease. All *p* values less than 0.05 were considered significant.

## Results

A total of 100 subjects were included in this study, as shown in Table 2. Fifty patients with steroid-induced ONFH and 50 healthy controls were included, with 25 males and 25 females in each group. The average age of the case group was 41.32 ± 13.37 years old, and that of the control group was 41.36 ± 12.45 years old. There were no significant differences in age, sex, total cholesterol (TC), or triglycerides (TGs) between the two groups. However, in patients with steroid-induced ONFH, the level of low-density lipoprotein-cholesterol (LDL-C) increased, while that of high-density lipoprotein-cholesterol (HDL-C) decreased. This state was consistent with the association analysis result between lipid metabolism indexes and steroid-induced ONFH [17]. The methylation status of the *OPG*, *RANK* and *RANKL* genes was determined by the Agena MassARRAY system. We implemented strict quality control on the methylation data and discarded unreliable samples and CpG units.

**Table 2:** Characteristics of the participants

Variable (s)	Case (n = 50)	Control (*n* = 50)	*p* value
Sex (N, %)			1.000 ^a^
Male	25 (25.0)	25 (25.0)	
Female	25 (25.0)	25 (25.0)	
Age, years (mean ± SD)	41.36 ± 12.45	41.32 ± 13.37	0.988 ^b^
Clinical stages			
Stage I-II	20 (40.0)		
Stage III-IV	30 (60.0)		
TC (mmol/L)	4.85 ± 1.05	4.80 ± 1.05	0.780 ^b^
TG (mmol/L)	1.77 ± 1.06	1.96 ± 1.12	0.323 ^b^
HDL-C (mmol/L)	1.07 ± 0.27	1.18 ± 0.30	< 0.001 ^b^
LDL-C (mmol/L)	2.64 ± 0.76	2.49 ± 0.71	0.015 ^b^

*TC* Total cholesterol, *TG* Triglycerides, *LDL-C* Low-density lipoprotein-cholesterol *LDL-C* High-density lipoprotein-cholesterol

*p* < 0.05 indicates statistical significance

^a^ Chi-squared test

^b^ Independent samples t test

The methylation status of the OPG, RANK, and RANKL genes in patients with steroid-related ONFH and control subjects.

**Fig. 2:**
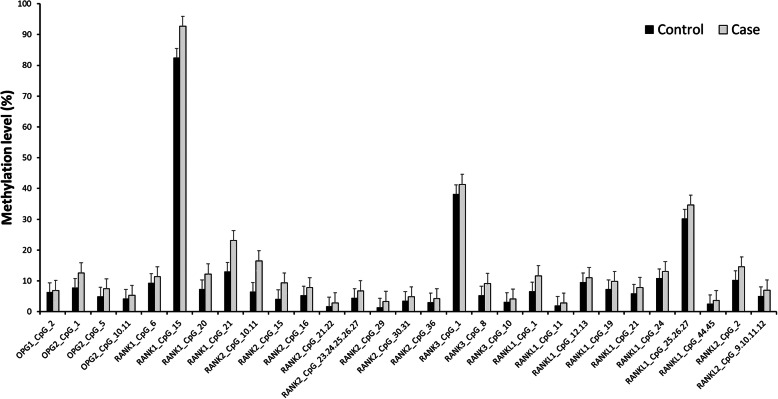
Intergroup differences in the methylation levels of CpG units. Compared with the control group, the methylation levels of 4 CpG units in *OPG*, 15 CpG units in *RANK* and 10 CpG units in *RANKL* were significantly increased

The methylation levels of the *OPG*, *RANK*, and *RANKL* genes in patients with steroid-related ONFH and control subjects are shown in Table 3. We found a CpG island (chr8:118952,332 ~ 118,952,550) in the *OPG* gene, in which 4 CpG units (*OPG*1_CpG_2, *OPG*2_CpG_1, *OPG*2_CpG_5, and *OPG*2_CpG_10.11) had a significantly higher methylation level than the control group. There are two CpG islands (chr18:62,325,388 ~ 62,326,216 and 62,326,241 ~ 62,326,536) in the *RANK* gene. Among them, the methylation level of 15 CpG units (*RANK*1_CpG_6, *RANK*1_CpG_15, *RANK*1_CpG_20, *RANK*1_CpG_21, *RANK*2_CpG_10.11, *RANK*2_CpG_15, *RANK*2_CpG_16, *RANK*2_CpG_21.22, *RANK*2_CpG_23.24.25.26.27, *RANK*2_CpG_29, *RANK*2_CpG_30.31, *RANK*2_CpG_36, *RANK*3_CpG_1, *RANK*3_CpG_8, and *RANK*3_CpG_10) was higher than that of the control group, and the difference was statistically significant. There was a CpG island (chr13:42,574,105 ~ 42,574,458) in the *RANKL* gene, and the methylation level of 10 CpG units (*RANKL*1_CpG_1, *RANKL*1_CpG_11, *RANKL*1_CpG_12.13, *RANKL*1_CpG_19, *RANKL*1_CpG_21, *RANKL*1_CpG_24, *RANKL*1_CpG_25.26.27, *RANKL*1_CpG_44.45, *RANKL*2_CpG_2, and *RANKL*2_CpG_9.10.11.12) in the control group was significantly lower than that of case group. Through the histogram comparison (Fig. 2), we can see a significant difference in CpG units between the two groups.

**Table 3:** Methylation levels of the *OPG*, *RANK* and *RANKL* genes

Gene_ID	Number of subjects		Methylation levels %		*p* value
	Control	Case	Control (mean ± SD)	Case (mean ± SD)	
*OPG*1_CpG_2	49	50	0.06 ± 0.01	0.07 ± 0.01	0.046 ^a^
*OPG*2_CpG_1	50	49	0.08 ± 0.09	0.13 ± 0.10	0.016 ^b^
*OPG*2_CpG_5	47	47	0.05 ± 0.04	0.07 ± 0.06	0.032 ^a^
*OPG*2_CpG_10.11	48	50	0.04 ± 0.02	0.05 ± 0.03	0.020 ^b^
*RANK*1_CpG_6	48	49	0.16 ± 0.04	0.09 ± 0.08	0.035 ^a^
*RANK*1_CpG_15	49	50	0.82 ± 0.25	0.93 ± 0.20	0.001 ^a^
*RANK*1_CpG_20	49	50	0.07 ± 0.09	0.12 ± 0.15	0.005 ^a^
*RANK*1_CpG_21	48	47	0.13 ± 0.17	0.23 ± 0.22	0.044 ^a^
*RANK*2_CpG_10.11	46	50	0.07 ± 0.04	0.17 ± 0.19	< 0.001 ^a^
*RANK*2_CpG_15	47	50	0.04 ± 0.04	0.09 ± 0.08	0.001 ^a^
*RANK*2_CpG_16	46	48	0.05 ± 0.03	0.08 ± 0.02	< 0.001 ^a^
*RANK*2_CpG_21.22	47	50	0.02 ± 0.01	0.03 ± 0.03	0.043 ^a^
*RANK*2_CpG_23.24.25.26.27	47	50	0.04 ± 0.02	0.07 ± 0.03	< 0.001 ^a^
*RANK*2_CpG_29	47	50	0.01 ± 0.02	0.03 ± 0.04	0.007 ^a^
*RANK*2_CpG_30.31	45	49	0.04 ± 0.02	0.05 ± 0.01	< 0.001 ^a^
*RANK*2_CpG_36	47	50	0.03 ± 0.03	0.04 ± 0.03	0.019 ^a^
*RANK*3_CpG_1	46	50	0.38 ± 0.09	0.41 ± 0.07	0.023 ^a^
*RANK*3_CpG_8	46	50	0.05 ± 0.03	0.09 ± 0.00	< 0.001 ^b^
*RANK*3_CpG_10	47	50	0.03 ± 0.02	0.04 ± 0.01	0.007 ^a^
*RANKL*1_CpG_1	43	49	0.07 ± 0.05	0.12 ± 0.11	0.009 ^b^
*RANKL*1_CpG_11	45	50	0.02 ± 0.03	0.03 ± 0.02	0.014 ^a^
*RANKL*1_CpG_12.13	44	49	0.10 ± 0.07	0.11 ± 0.03	0.004 ^a^
*RANKL*1_CpG_19	43	49	0.07 ± 0.15	0.10 ± 0.14	0.001 ^a^
*RANKL*1_CpG_21	44	50	0.06 ± 0.08	0.08 ± 0.14	0.041 ^a^
*RANKL*1_CpG_24	46	50	0.11 ± 0.15	0.13 ± 0.16	0.018 ^a^
*RANKL*1_CpG_25.26.27	45	50	0.30 ± 0.19	0.35 ± 0.15	< 0.001 ^a^
*RANKL*1_CpG_44.45	45	50	0.03 ± 0.01	0.04 ± 0.01	< 0.001 ^b^
*RANKL*2_CpG_2	48	50	0.10 ± 0.06	0.15 ± 0.09	0.016 ^b^
*RANKL*2_CpG_9.10.11.12	48	50	0.05 ± 0.04	0.07 ± 0.02	< 0.001 ^a^

*p* < 0.05 indicates statistical significance

^a^ Mann-Whitney U test

^b^ Independent samples t test

**Fig. 3:**
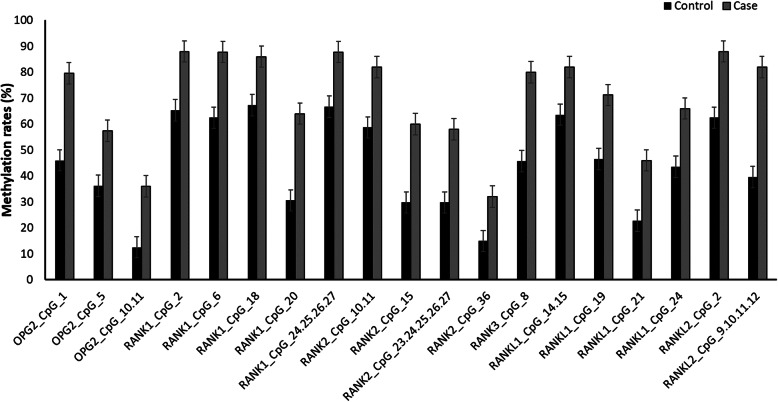
Intergroup differences in the methylation rates of CpG units. Compared with the control group, the methylation rates of 3 CpG units in *OPG*, 10 CpG units in *RANK* and 6 CpG units in *RANKL* were significantly increased

The differences in the methylation rates of the *OPG*, *RANK*, and *RANKL* genes between patients and control subjects are listed in Table 4. Compared with control subjects, the methylation rate of 3 CpG units (*OPG*2_CpG_1, *OPG*2_CpG_5, and *OPG*2_CpG_10.11) in the *OPG* gene, 10 CpG units (*RANK*1_CpG_2, *RANK*1_CpG_6, *RANK*1_CpG_18, *RANK*1_CpG_20, *RANK*1_CpG_24.25.26.27, *RANK*2_CpG_10.11, *RANK*2_CpG_15, *RANK*2_CpG_23.24.25.26.27, *RANK*2_CpG_36, and *RANK*3_CpG_8) in the *RANK* gene and 6 CpG units (*RANKL*1_CpG_14.15, *RANKL*1_CpG_19, *RANKL*1_CpG_21, *RANKL*1_CpG_24, *RANKL*2_CpG_2, and *RANKL*2_CpG_9.10.11.12) in the *RANKL* gene of patients with steroid-related ONFH was significantly increased (Fig. 3).

**Table 4:** Relationship between methylation rates and steroid-related osteonecrosis of femoral head

Gene_ID	Group	Number of subjects		*p* value ^a^
		Non-Meth.	Meth.	
*OPG*2_CpG_1	Control	27	23	0.001
	Case	10	39	
*OPG*2_CpG_5	Control	30	17	0.039
	Case	20	27	
*OPG*2_CpG_10.11	Control	42	6	0.007
	Case	32	18	
*RANK*1_CpG_2	Control	17	32	0.008
	Case	6	44	
*RANK*1_CpG_6	Control	18	30	0.004
	Case	6	43	
*RANK*1_CpG_18	Control	16	33	0.028
	Case	7	43	
*RANK*1_CpG_20	Control	34	15	0.001
	Case	18	32	
*RANK*1_CpG_24.25.26.27	Control	16	32	0.013
	Case	6	43	
*RANK*2_CpG_10.11	Control	19	27	0.012
	Case	9	41	
*RANK*2_CpG_15	Control	33	14	0.003
	Case	20	30	
*RANK*2_CpG_23.24.25.26.27	Control	33	14	0.005
	Case	21	29	
*RANK*2_CpG_36	Control	40	7	0.048
	Case	34	16	
*RANK*3_CpG_8	Control	25	21	< 0.001
	Case	10	40	
*RANKL*1_CpG_14.15	Control	16	28	0.044
	Case	9	41	
*RANKL*1_CpG_19	Control	23	20	0.015
	Case	14	35	
*RANKL*1_CpG_21	Control	34	10	0.018
	Case	27	23	
*RANKL*1_CpG_24	Control	26	20	0.027
	Case	17	33	
*RANKL*2_CpG_2	Control	18	30	0.003
	Case	6	44	
*RANKL*2_CpG_9.10.11.12	Control	29	19	< 0.001
	Case	9	41	

Non-Meth Non-Methylation; Meth Methylation

*p* < 0.05 indicates statistical significance

^a^ chi-square test

Through the above comparative analysis, we found that the methylation status of the *OPG*, *RANK*, and *RANKL* genes was indeed different between patients and healthy subjects. To further study the effect of methylation on steroid-induced ONFH, we divided the data into two groups with hypermethylation and hypomethylation based on the average level of methylation in the control group. Logistic regression analysis was then performed. The results in Table 5 show that in the *OPG* gene, hypermethylation levels at 2 CpG sites (*OPG*2_CpG_1 and *OPG*2_CpG_5) increased the risk of steroid-induced ONFH. In the *RANK* gene, 12 hypermethylated CpG units (*RANK*1_CpG_6, *RANK*1_CpG_15, *RANK*1_CpG_20, *RANK*2_CpG_10.11, *RANK*2_CpG_15, *RANK*2_CpG_16, *RANK*2_CpG_21.22, *RANK*2_CpG_23.24.25.26.27, *RANK*2_CpG_29, *RANK*2_CpG_36, *RANK*3_CpG_8, and *RANK*3_CpG_10) were risk factors for steroid-induced ONFH. In the *RANKL* gene, the increase in the methylation level of 10 CpG units (*RANKL*1_CpG_1, *RANKL*1_CpG_12.13, *RANKL*1_CpG_14.15, *RANKL*1_CpG_19, *RANKL*1_CpG_21, *RANKL*1_CpG_24, *RANKL*1_CpG_25.26.27, *RANKL*1_CpG_44.45, *RANKL*1_CpG_46, and *RANKL*2_CpG_9.10.11.12) also increased the risk of the disease. By dividing the *OPG*, *RANK*, and *RANKL* genes into high and low methylation levels, we found that the tendency toward hypermethylation is more meaningful for the risk of steroid-induced ONFH. This step is only a further extension of the correlation between the methylation level and steroid-induced ONFH. However, it must be mentioned that this processing method may cause a loss of freedom to a certain extent.

**Table 5:** Relationship between methylation and steroid-associated osteonecrosis of femoral head

Gene_ID	OR	95% CI		*p* value
		lower limit	upper limit	
*OPG*2_CpG_1	4.62	1.95	10.95	0.001
*OPG*2_CpG_5	3.45	1.32	9.02	0.005
*RANK*1_CpG_6	4.83	1.64	14.25	0.004
*RANK*1_CpG_15	4.67	1.82	12.01	0.001
*RANK*1_CpG_20	4.25	1.80	10.05	0.001
*RANK*2_CpG_10.11	4.76	1.95	11.65	0.001
*RANK2*_CpG_15	4.17	1.69	10.29	0.002
*RANK*2_CpG_16	3.71	1.30	10.59	0.014
*RANK*2_CpG_21.22	2.90	1.19	7.05	0.019
*RANK*2_CpG_23.24.25.26.27	3.43	1.45	8.10	0.005
*RANK*2_CpG_29	3.48	1.36	8.91	0.009
*RANK*2_CpG_36	2.95	1.27	6.86	0.012
*RANK*3_CpG_8	6.56	2.54	16.93	< 0.001
*RANK*3_CpG_10	3.91	1.65	9.27	0.002
*RANKL*1_CpG_1	12.66	4.10	39.11	< 0.001
*RANKL*1_CpG_12.13	3.57	1.42	8.98	0.007
*RANKL*1_CpG_14.15	3.10	1.30	7.38	0.011
*RANKL*1_CpG_19	6.92	2.66	18.00	< 0.001
*RANKL*1_CpG_21	3.05	1.30	7.12	0.010
*RANKL*1_CpG_24	2.44	1.05	5.64	0.038
*RANKL*1_CpG_25.26.27	5.89	2.32	14.93	< 0.001
*RANKL*1_CpG_44.45	3.84	1.53	9.62	0.004
*RANKL*1_CpG_46	3.74	1.40	9.97	0.008
*RANKL*2_CpG_9.10.11.12	3.18	1.31	7.73	0.011

*OR* odds ratio; *CI* confidence interval

*p* < 0.05 indicates statistical significance

*p* values were calculated by logistic regression adjusted for age and sex

### Predictive indicators of steroid-related ONFH

**Fig. 4:**
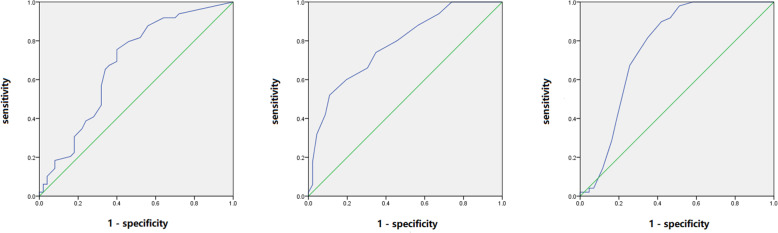
ROC analysis of the methylated CPG units to diagnose patients with steroid-related ONFH. *OPG*2_CpG_1 (**A**), *RANK*3_CpG_8 (**B**) and *RANKL*1_CpG_1 (**C**) were the most valuable sites in the diagnosis of steroid-associated ONFH among the three genes

To investigate the applicability of methylation of the *OPG*, *RANK*, and *RANKL* genes as potential diagnostic biomarkers for steroid-related ONFH, follow-up ROC curve analysis was performed on the above data (Table 6). In the *OPG* gene, the area under the curve (AUC) values of 3 CpG units (*OPG*2_CpG_1, *OPG*2_CpG_5 and *OPG*2_CpG_10.11) were greater than 0.5, of which *OPG*2_CpG_1 (AUC: 0.679 95% CI: 0.57–0.79 *p* = 0.002) had the highest diagnostic value (Fig. 4A). In the *RANK* gene, the AUC values of 15 CpG units (*RANK*1_CpG_6, *RANK*1_CpG_15, *RANK*1_CpG_20, *RANK*1_CpG_21, *RANK*2_CpG_10.11, *RANK*2_CpG_15, *RANK*2_CpG_16, *RANK*2_CpG_21.22, *RANK*2_CpG_23.24.25.26.27, *RANK*2_CpG_29, *RANK*2_CpG_30.31, *RANK*2_CpG_36, *RANK*3_CpG_1, *RANK*3_CpG_8 and *RANK*3_CpG_10) were greater than 0.5, of which *RANK*3_CpG_8 (AUC: 0.778 95% CI: 0.69–0.87 *p* < 0.001) was more significant in predicting disease (Fig. 4B). In the *RANKL* gene, there were 10 CpG units (*RANKL*1_CpG_1, *RANKL*1_CpG_11, *RANKL*1_CpG_12.13, *RANKL*1_CpG_19, *RANKL*1_CpG_21, *RANKL*1_CpG_24, *RANKL*1_CpG_25.26.27, *RANKL*1_CpG_44.45, *RANKL*2_CpG_2 and *RANKL*2_CpG_9.10.11.12) with AUC values greater than 0.5, of which *RANKL*1_CpG_1 (AUC: 0.764 95% CI: 0.66–0.87 *p* < 0.001) had the highest diagnostic value (Fig. 4C).

**Table 6:** Area under the curve of the methylation sites

Gene_ID	Area	Sem^a^	Youden’s index	95% CI		*p* value ^b^
				lower limit	upper limit	
*OPG*2_CpG_1	0.679	0.055	0.355	0.57	0.79	0.002
*OPG*2_CpG_5	0.628	0.059	0.319	0.51	0.74	0.032
*OPG*2_CpG_10.11	0.622	0.057	0.235	0.51	0.73	0.037
*RANK*1_CpG_6	0.624	0.057	0.253	0.51	0.74	0.036
*RANK*1_CpG_15	0.669	0.055	0.330	0.56	0.78	0.004
*RANK*1_CpG_20	0.662	0.055	0.334	0.56	0.77	0.005
*RANK*1_CpG_21	0.619	0.059	0.324	0.50	0.74	0.045
*RANK*2_CpG_10.11	0.750	0.051	0.450	0.65	0.85	< 0.001
*RANK*2_CpG_15	0.697	0.053	0.394	0.59	0.80	0.001
*RANK*2_CpG_16	0.760	0.050	0.442	0.66	0.86	< 0.001
*RANK*2_CpG_21.22	0.617	0.057	0.226	0.51	0.73	0.047
*RANK*2_CpG_23.24.25.26.27	0.718	0.051	0.352	0.62	0.82	< 0.001
*RANK*2_CpG_29	0.655	0.056	0.295	0.55	0.76	0.008
*RANK*2_CpG_30.31	0.688	0.055	0.301	0.58	0.80	0.002
*RANK*2_CpG_36	0.636	0.057	0.256	0.53	0.75	0.021
*RANK*3_CpG_1	0.635	0.058	0.315	0.52	0.75	0.023
*RANK*3_CpG_8	0.778	0.047	0.411	0.69	0.87	< 0.001
*RANK*3_CpG_10	0.699	0.053	0.318	0.59	0.80	0.001
*RANKL*1_CpG_1	0.764	0.054	0.479	0.66	0.87	< 0.001
*RANKL*1_CpG_11	0.645	0.057	0.256	0.53	0.76	0.015
*RANKL*1_CpG_12.13	0.672	0.059	0.327	0.58	0.79	0.004
*RANKL*1_CpG_19	0.697	0.056	0.420	0.59	0.81	0.001
*RANKL*1_CpG_21	0.622	0.059	0.271	0.51	0.74	0.042
*RANKL*1_CpG_24	0.640	0.058	0.302	0.53	0.75	0.019
*RANKL*1_CpG_25.26.27	0.710	0.056	0.411	0.60	0.82	< 0.001
*RANKL*1_CpG_44.45	0.734	0.052	0.396	0.63	0.84	< 0.001
*RANKL*2_CpG_2	0.629	0.057	0.278	0.52	0.74	0.027
*RANKL*2_CpG_9.10.11.12	0.738	0.052	0.438	0.64	0.84	< 0.001

Sem Standard error of mean; CI Confidence index

*p* < 0.05 indicates statistical significance

^a^ Under non-parametric assumptions

^b^ Null hypothesis: real area = 0.5

## Discussion

DNA methylation is an extremely abundant chemical modification. Its abnormal changes (including hypermethylation or hypomethylation) are related to gene expression and contribute to the development of diseases. At present, most of the research on DNA methylation has focused on CpG islands or short DNA fragments that are essential for transcriptional regulation and rich in high CpG sites, such as gene promoter regions [18, 19]. When these CpG sites that specifically bind to transcription factors are hypermethylated, the combination of promoters and transcription factors is reduced, thereby silencing or inhibiting gene transcription and expression. Many studies on the methylation of target genes have clarified the relationship between abnormal methylation and disease [20, 21]. In recent years, increasing evidence has shown that DNA methylation modification also affects the pathogenesis of nontraumatic ONFH. Some related methylation genes and loci have also been reported, such as the *ABCB1* gene, *RUNX2* gene, *FZD1* gene, and *CARS* gene.

In previous studies, the methylation status of the *OPG*/*RANK*/*RANKL* system was closely related to bone metabolic diseases. For example, Delgado-Calle, J. et al. [22] studied the role of DNA methylation in regulating the *OPG/RANKL* system in human bone. The results showed that in HEK-293 and HOS-TE85 cells, the degree of methylation in the CPG-enriched region of the *OPG* gene was higher, and its expression was reduced. *RANKL* was hypermethylated in the osteoblast cell line HOS-TE85 and renal-derived HEK-293 cells, and its mRNA expression was decreased. This suggests that DNA methylation inhibits the expression of *OPG* and *RANKL* genes. Wang Peng et al. [23] found that in the bone tissue of osteoporotic fractures, the CpG methylation status of the *RANKL* gene promoter was remarkably reduced, and the expression was significantly increased. However, for the *OPG* gene, a higher degree of methylation and a lower expression level were found. It has not been reported whether methylation changes in this pathway also contribute to the occurrence of steroid-related ONFH. Therefore, we investigated the methylation status of CpG islands in the *OPG*, *RANK*, and *RANKL* genes in patients with steroid-related ONFH.

In the case group of this study, we found that the methylation levels of 4 CpG units in the *OPG* gene, 15 CpG units in the *RANK* gene, and 10 CpG units in the *RANKL* gene were significantly higher than those in the healthy control group (*p* < 0.05). In the control group, the methylation rates of 3 CpG units in the *OPG* gene, 10 CpG units in the *RANK* gene, and 6 CpG units in the *RANKL* gene were lower than those in the case group (*p* < 0.05). Obviously, the degree of methylation of the *OPG*, *RANK,* and *RANKL* genes in patients with steroid-induced ONFH is different from that of healthy people. Hypermethylation is more significant in the pathogenesis of the disease. In addition, we found that two hypermethylated CpG units in the *OPG* gene, twelve hypermethylated CpG units in the *RANK* gene, and ten hypermethylated CpG units in the *RANKL* gene increased the risk of steroid-related ONFH (*p* < 0.05). To avoid the possible influence of age (the levels of methylation may be higher with age [24]) and sex on methylation, the two groups in this study were matched. The logistic regression analysis has also been revised. Because the *OPG*/*RANK*/*RANKL* pathway is also very important for the epigenetic regulation of obesity [25], this may also be one of the reasons for the difference in high- and low-density proteins between the two groups. Because of the small sample size, we did not conduct further stratified analysis on the course of the disease or physical indicators (such as age, sex, and BMI), which would reduce the efficiency of the test.

In other steroid diseases, methylation of the *OPG, RANK,* and *RANKL* genes has not been reported. However, the possible effect of steroids on gene methylation needs to be considered when designing case and control groups. All patients receiving systemic steroid medication are at risk of steroid-induced ONFH. Among the types of patients, SLE with ONFH is more common (approximately 30%), followed by inflammatory diseases (such as pneumonia, nephritis, hepatitis), blood system diseases (for example, thrombocytopenic purpura, aplastic anaemia), connective tissue diseases, and other diseases (for instance, eye diseases and skin diseases) [26]. Most of the patients included in this study were treated with steroids due to inflammatory disease, which resulted in ONFH. There were no healthy people receiving steroid treatment. The control group included healthy subjects who were widely used in most of the studies, rather than patients receiving steroid treatment with other conditions [27]. The reason is that even if the patients with SLE and ONFH, which accounted for the highest proportion of the disease, were taken as the control group, the resulting statistical conclusion could only prove the difference between the two diseases. At present, some studies have set up multiple control groups, such as the healthy group, SONFH group, and SLE group at the same time [28], hoping to reduce the interference of hormones on disease. However, whether this approach can completely counteract the effects of hormones on disease remains in question. In addition, the association between the degree of DNA methylation and hormone levels has not been established, mainly because of the lack of accurate statistics on hormone intake in the case group.

The regulatory effects of *OPG, RANK* and *RANKL* on osteoclasts are self-evident. The hypermethylation of CpG islands in the *OPG*, *RANK*, and *RANKL* genes may reduce the expression of related mRNAs, thereby downregulating the *OPG*/*RANK*/*RANKL* signalling pathway and leading to osteoclast dysfunction in patients with steroid-related ONFH. As a result, osteoclasts in the bone marrow of the femoral head increase, bone resorption decreases, and the structure of bone trabeculae becomes thin and disordered. Bone density decreases, leading to severe osteoporosis. The femoral head is prone to compression fractures, resulting in collapse and necrosis. Changes in the methylation status of this pathway may also lead to disorders of endothelial cell metabolism and function. On the one hand, the damaged microvascular endothelial cells can form micro-thromboses and block local blood vessels, leading to ischaemia and necrosis of the femoral head in the innervated area. On the other hand, a large number of reactive oxygen species are produced, which reduces the synthesis of vasodilatory substances and further aggravates bone cell damage. This intensifies the progression of steroid-related ONFH [10, 29].

Studies have shown that quantitative analysis of cytosine methylation can identify molecular markers in diseases [30]. Specific hypermethylated CpG sites can be used to diagnose diseases [31, 32]. To further study the applicability of *OPG*, *RANK*, and *RANKL* gene methylation levels as potential diagnostic biomarkers for steroid-related ONFH, ROC analysis was performed. The results showed that the AUC values of 3 CpG units in the *OPG* gene, 15 CpG units in the *RANK* gene, and 10 CpG units in the *RANKL* gene were greater than 0.5. Among them, *RANK*2_CpG_10.11, *RANK*2_CpG_16, *RANK*2_CpG_23.24.25.26.27, *RANK*3_CpG_8, *RANKL*1_CpG_1, *RANKL*1_CpG_25.26.27, *RANKL*1_CpG_44.45, and *RANKL*2_CpG_9.10.11.12 are the CpG units with the most diagnostic value, which may have a certain significance for the early prediction of steroid-related ONFH.

In this experiment, we selected the patient’s blood for testing, not bone tissue. Is there a difference in methylation between the two? Ebrahimi P et al. [33] believed that peripheral blood is a viable substitute for bone tissue in DNA methylation studies. However, Walton E et al. [34] suggested that there was a lack of correspondence between DNA methylation in blood and tissues. Most DNA methylation markers in peripheral blood cannot reliably predict DNA methylation status. In this experiment, the sample we selected was blood, which may not be as sensitive as femoral head tissue in investigating the cause of steroid-related ONFH.

Methylation itself is a reversible chemical modification of DNA. Methylated cytosine can be converted back into cytosine through active or passive demethylation. Given this characteristic, we not only hope to diagnose the disease by identifying DNA methylation, but also wish to treat the disease by changing the DNA methylation. Therefore, can diseases be treated by changing the status of DNA methylation? Some studies have shown that regulating the degree of methylation of the target gene, has a positive therapeutic effect on diseases. For example, DNA methylation inhibitors have become the principal means to treat certain haematological malignancies (such as myelodysplastic syndrome, chronic myelomonocytic leukaemia, and acute myeloid leukemia) [35, 36]. Some drugs can treat kidney disease by inhibiting DNA methylation (such as 5-azacytidine and decitabine) or activating DNA demethylation (such as hydralazine) [37]. In orthopaedic diseases, DNA methylation is also expected to become a future treatment and diagnosis target for RA [38]. The author believes that the treatment of disease by changing the DNA methylation status is very promising. However, how DNA methylation acts on related proteins in steroid-related ONFH and treats the disease still requires in-depth exploration and further research.

It must be pointed out that our research has certain limitations. First, in this study, only an association between DNA methylation and steroid-related ONFH was studied, not causality. In particular, we did not detect the expression levels of the three genes. The results of DNA methylation analysis by MassARRAY require further validation. Second, abnormal DNA methylation is not confined to CpG islands. Methylation changes in other sites (such as the CpG shore and CpG shelve) should not be ignored. Third, the small sample size of our research limits the generalization of the results. In subsequent studies, we will use more reasonable test methods on larger samples to verify the above results.

In summary, our results show that the methylation status of the *OPG*, *RANK*, and *RANKL* genes in steroid-related ONFH patients is different from that of normal controls. The hypermethylation of some CpG units increases the risk of steroid-related ONFH. Detecting the methylation of the CpG sites of the above genes, it has certain significance for the early diagnosis of steroid-related ONFH.

## Conclusion

In this study, the Agena MassARRAY system was used to analyse the methylation status of the *OPG*, *RANK*, and *RANKL* genes in the blood of 50 healthy subjects and 50 patients. Significant changes in DNA methylation were observed. Many hypermethylated CpG units are associated with the risk of steroid-related ONFH. Our results indicate an association between the methylation of the *OPG*, *RANK*, and *RANKL* genes and steroid-related ONFH.

## Data Availability

The datasets used during the current study are available from the corresponding author on reasonable request.

## Abbreviations

OPG: Osteoprotegerin
RANK: Receptor activator of nuclear factor-κ B
RANKL: Receptor Activator of Nuclear Factor-κ B Ligand
ONFH: Osteonecrosis of the femoral head
CPG: Cytosine-phosphate-guanine
ROC: Receiver operating characteristic
ARCO: Association Research Osteocirculatory System
MRI: Magnetic resonance imaging
CT: Computer tomography
PCR: Perform polymerase chain reaction (PCR)
OR: Odds ratio
CI: Confidence interval
